# Safe Use of Screen Time Among Adolescents: A Randomized Controlled Study of the Efficacy of Yoga

**DOI:** 10.7759/cureus.71335

**Published:** 2024-10-12

**Authors:** Manisha Mona, Sony Kumari, Nitin Anand, Manoj Sharma

**Affiliations:** 1 Yoga and Management, Swami Vivekananda Yoga Anusandhana Samsthana (S-VYASA) University, Bengaluru, IND; 2 Management, Swami Vivekananda Yoga Anusandhana Samsthana (S-VYASA) University, Bengaluru, IND; 3 Clinical Psychology, National Institute of Mental Health and Neurosciences, Bengaluru, IND

**Keywords:** adolescents, mental health, screen time, smartphone addiction, yoga

## Abstract

Background: For students between the ages of 11 and 18, the estimated daily exposure to electronic media is around 1.5 hours, suggesting a growing trend in the amount of time spent in front of a screen. Increasing screen time use is related to sleep, mental health, cognitive, emotional, and behavioral disorders, unhealthy diets, depressive symptoms, poor quality of life, and substance-behavioral addictions.

Methods: The current study is a parallel-group, simple randomized controlled trial (RCT). A total of 100 participants were randomized into a yoga group (YG) (n=50) and an attentional control group (CG) (n=50) by using an online randomizer. The YG received yoga practice, whereas the partially active CG received an educational program on digital hygiene. Students were assessed for screen time use, Smartphone Addiction Scale-Short Version (SAS-SV), general well-being, depressive symptoms, and generalized anxiety following 12 weeks of intervention.

Results: The yoga group showed a significant decrease in the scores of SAS-SV, generalized anxiety, depressive symptoms, and screen time use after the intervention (p<0.001, p<0.05, p<0.01, and p<0.001, respectively). Furthermore, there was a statistically significant decrease in screen time use for all modes of total weekdays, weeknights, and weekends. Additionally, in the yoga group, the majority of the sub-factors of screen time use showed statistically significant changes; time spent on television showed a constant decrease in all modes such as weekdays, weeknights, and weekends.

Conclusion: The result of the current study suggests that yoga practice has a positive influence on screen usage behavior and associated health-related complications. More studies are required to understand if yoga practice can help mitigate the pleasure-seeking behavior that drives the excessive usage of screens.

## Introduction

Childhood today is being shaped by electronic devices of the new era, which is brought by flat panel displays, mobile computing capabilities, and high-speed internet connections. It is reported that 69% of kids own a cell phone, and by the time they are 12 years old, 85% of them use screens for recreation [1]. For those between the ages of 11 and 18, the estimated daily exposure to electronic media is around 1.5 hours, suggesting a growing trend in the amount of time spent in front of a screen [2].

Screen time use, which is the overall amount of time spent using electronic devices, including computers, smartphones, and television screens, is a growing concern for children, adolescents, and young adults. Prolonged screen exposure is associated with poor sleep quality, cardiovascular risk, obesity, stress management issues, and insulin resistance [3,4]. Apart from that, it can also result in cognitive decline [5], memory issues, slower information processing, weaker impulse control, anti-social tendencies, emotional desensitization, emotion dysregulation, anxiety, and depression [6,7]. Children who engage in greater amounts of screen time (TV viewing and video gaming) are more likely to consume alcohol and solvents than children who engage in less screen time [8]. Furthermore, new research indicates that some of the effects of screen time on the brain are comparable to those observed in adults who exhibit mild cognitive impairments in the early stages of dementia [6]. These impairments include diminished social functioning, self-care, cognition, orientation, the acquisition of recent memories (anterograde amnesia), and the ability to recall memories (retrograde amnesia). Chronic sensory overstimulation (i.e., excessive screen time) during brain development increases the risk of accelerated neurodegeneration in adulthood (i.e., amnesia and early-onset dementia) [4]. These mental health concerns linked to screen time are extremely crucial.

To tackle this growing concern, the World Health Organization (WHO) advises preventing excessive screen time or gaming by identifying the warning signs and acting immediately [9]. With the increased concern about excessive screen time, some countries around the world have placed some regulations on its usage. For instance, Taiwanese parents must legally monitor their children's screen time, while China and South Korea equate excessive screen use with harmful habits such as smoking and drinking [10]. Thus, it is very clear that excessive screen time among children and adolescents is a commonly shared concern across the globe.

Knowing the impact of excessive usage of screens, an effective intervention strategy must be developed to mitigate the long-term potential impact on adolescents. Yoga practice can be one such intervention.

Yoga is a well-known lifestyle-based intervention worldwide. It has gained popularity as a holistic approach to addressing various behavioral and psychological issues. Yoga practices, including postures, breath control, and mindfulness meditation, have been reported to reduce stress, improve emotional regulation, and enhance self-awareness [11]. Incorporating yoga-based interventions may address the variables associated with excessive screen time use, such as depression and anxiety. The practice of yoga has been demonstrated to reduce stress, anxiety, and depression [12]. It induces a sense of calmness, well-being, stress tolerance, and mental focus, which can minimize depression, anxiety, stress, and rumination. Yoga practice may bring these positive changes by altering the perception and appraisal of stressors and physiological reactions [13]. As per literature reviews, no randomized controlled trial (RCT) study on yoga has explored the effect of an integrated yoga approach on screen time use. Hence, the current study intends to assess the impact of an integrated approach of yoga to address excessive screen time use and its associated complications such as depressive symptoms, anxiety, physical health problems, and well-being among students.

## Materials and methods

Study design and participants

**Figure 1:**
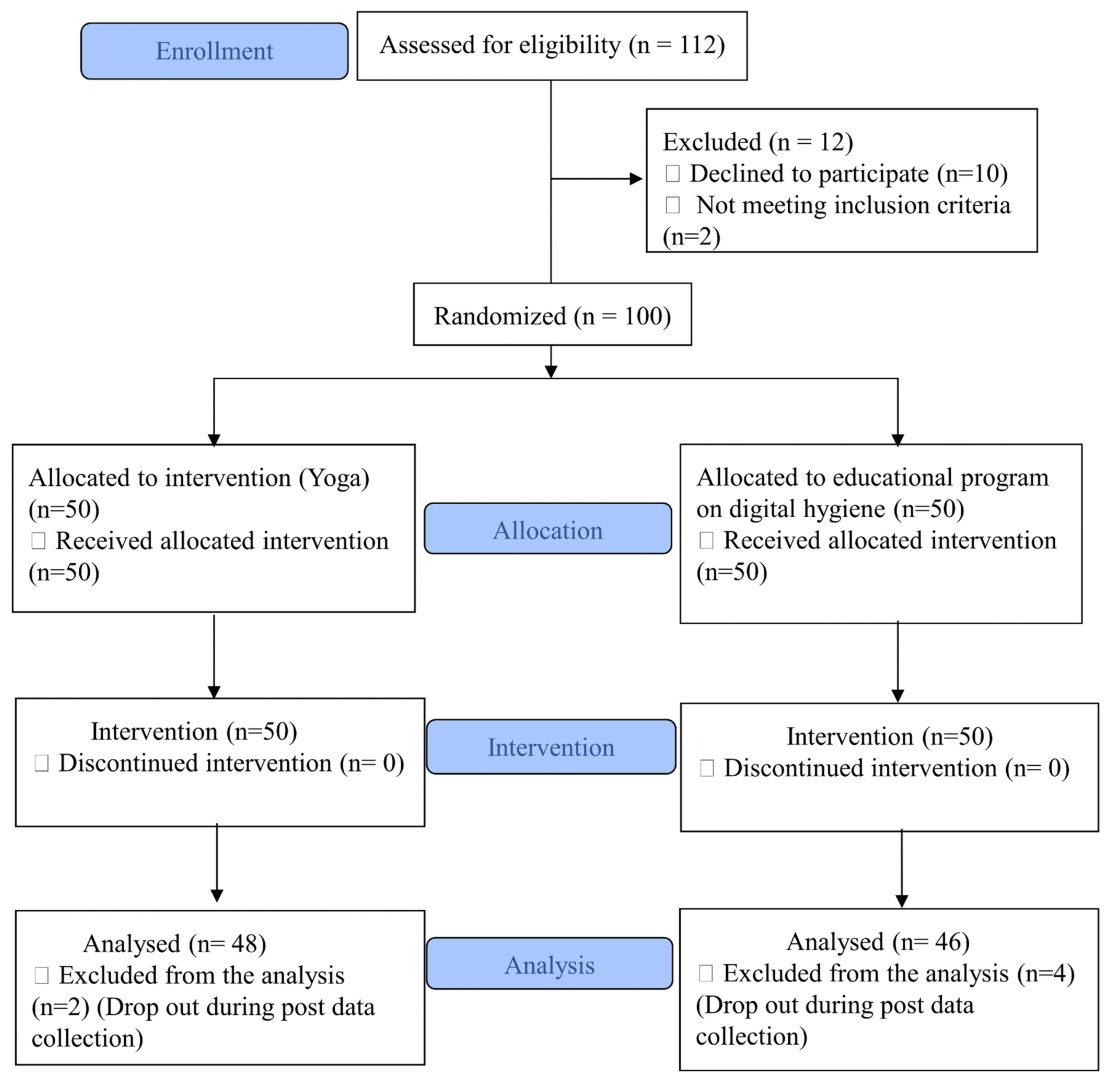
CONSORT flow diagram The CONSORT flow diagram depicts the recruitment and screening process of the participants included in the study. CONSORT: Consolidated Standards of Reporting Trials

A total of 100 participants, aged between 13 and 17 years, were recruited for the study. All were school students. An online randomizer (https://www.randomlists.com/list-randomizer) was used to randomize the participants into two groups [14]. Fifty participants were assigned to the yoga group (YG) (age±standard deviation (SD): 13.44±0.61 years) and 50 to the control group (CG) (age±SD: 13.54±0.64 years). Hence, the study was a parallel-group, simple randomized controlled trial (RCT). The sample size was calculated using G-Power software (Franz Faul, Universitat Kiel, Germany) by fixing the alpha at 0.05, power at 0.95, and effect size at 0.86 based on our previous pilot study (submitted for publication elsewhere) using the variable Smartphone Addiction Scale-Short Version (SAS-SV). The G-Power software generated a sample size of 64; considering the possibility of dropouts, 100 participants were recruited for the study. There were two dropouts from the yoga group and four from the control group. Therefore, in the final analysis, a total of 94 participant data were used. Figure 1 shows the Consolidated Standards of Reporting Trials (CONSORT) flow diagram of the randomization process.

The participants were recruited by contacting several educational institutions. The inclusion criteria included those who could read and write in English and those who were willing to participate in the study, and all genders were included in the study. Potential participants were excluded from the study if they had surgery in the last six months, had no digital gadgets, had been doing yoga for the last six months, and had a screen time of less than two hours.

Ethical considerations

The study was approved by the Institutional Ethical Committee (IEC) of Swami Vivekananda Yoga Anusandhana Samsthana (S-VYASA) University (IEC number: RES/IEC-SVYASA/224/2022). The study was conducted according to the Declaration of Helsinki guidelines on ethical conduct involving human subjects. An informed consent form was obtained from all the participants. The current study was registered in the Clinical Trial Registry of the Government of India (CTRI/2022/05/042360).

Intervention

The yoga protocol adopted in the study consisted of asanas, pranayama (breathing practices), kriya, yogic games, and relaxation techniques. The duration of the yoga intervention was three months (January 6 to April 5, 2023), 45 minutes a day, and three days a week.

The educational program on digital hygiene issued by the University Grants Commission (UGC) (India) was provided to the control group for the same duration as the yoga group.

Outcome measures

Screen Time Use Questionnaire (STQ)

This questionnaire was used to assess the screen time use of the participants. It is a self-reported questionnaire with 18 items; each item asks about the hours and minutes of usage. It gives usage in terms of five devices: television, TV-connected devices, laptop, smartphone, and tablet. It provides frequency and duration of use. The screen time use was divided into three types according to STQ: light users (median: seven hours/day), moderate users (median: 11.25 hours/day), and heavy users (median: 17.5 hours/day) (test-retest reliability (inter-class correlation coefficients (ICCs)): 0.50-0.90, all: <0.000). The standard error of the mean (SEM) values for all screen types were large [15].

Smartphone Addiction Scale-Short Version (SAS-SV)

The Smartphone Addiction Scale is a 10-item questionnaire. The scores on this scale are measured on a 6-point Likert scale. The six factors analyzed by this questionnaire were daily life disturbance, positive anticipation, withdrawal, cyberspace-oriented relationship, overuse, and tolerance. The final score is measured by summing all the items in the scale. The SAS-SV has shown good reliability and validity for the assessment of smartphone addiction. The Cronbach's alpha for the scale is 0.91 [16].

World Health Organization (WHO)-5 Well-Being Index (WHO, 1998)

This is a widely used five-item questionnaire that assesses subjective psychological well-being. Each item of the questionnaire consists of a rating based on a Likert scale from 0 to 5 that is responded to in relation to the past two weeks of duration. The WHO-5 is considered a purely generic scale to assess well-being, with a weighted sensitivity of 0.86 and a specificity of 0.81. A WHO-5 cutoff score of ≤50 is recommendable when screening for clinical depression. The scale has shown high reliability, with adequate construct and criterion validity, identification, and a good measure for screening depression and used as an outcome measure for interventional studies [17].

Patient Health Questionnaire-9 (PHQ-9)

The Patient Health Questionnaire-9 (PHQ-9) has demonstrated good internal consistency, indicating that the questionnaire items measure the same underlying construct of depression. High internal consistency and reliability have been reported in various populations, including primary care patients, general adult populations, and adolescents [18].

Generalized Anxiety Disorder-7 (GAD-7)

Generalized Anxiety Disorder-7 (GAD-7) for adolescents has shown good validity in measuring anxiety symptoms. Studies have demonstrated a strong correlation between GAD-7 scores and other established anxiety measures, supporting its convergent validity. It has also been validated against clinician interviews for diagnosing generalized anxiety disorder in adolescents, indicating good diagnostic accuracy [19].

Statistical analysis

Statistical test was performed using a statistical analysis software package IBM SPSS Statistics version 21.0 software (IBM Corp., Armonk, NY). The normality of data was assessed using the Kolmogorov-Smirnov test. For SAS-SV and WHO-5, the data were normally distributed. There were two groups (between factors, i.e., yoga and control) and two states (within factors, i.e., pre and post). Hence, to avoid multiple comparison type I errors, repeated measures analysis of variance (ANOVA) was conducted for the analysis of the aforementioned parameters. For screen time (PHQ-9 and GAD-7), a non-parametric test was conducted, i.e., Wilcoxon signed-rank test (within-group comparison) and Mann-Whitney U test (between-group comparison) as data were not normally distributed for these parameters. The results were considered statistically significant if the p ≤ 0.05. Additionally, effect size and percentage change were calculated for all the parameters. The formula used to calculate the percentage change was as follows: ((post/pre) × 100-100).

## Results

**Figure 2:**
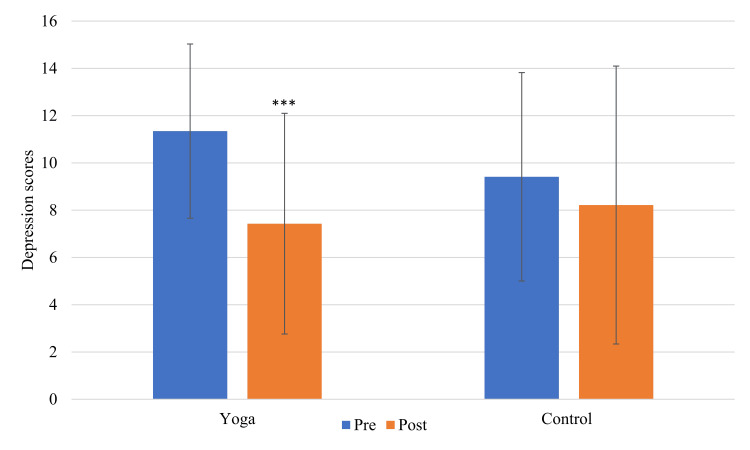
Graph of within-group difference for depressive symptoms ***p<0.001

**Figure 3:**
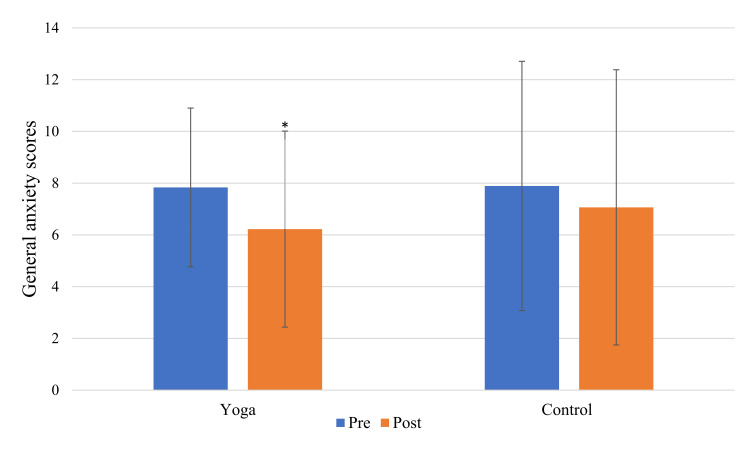
Graph of within-group difference for general anxiety symptoms *p<0.05

The demographic characteristics of the participants are presented in Table 1. The yoga group showed a significant decrease in the scores of SAS-SV, depressive symptoms, generalized anxiety, and screen time after the intervention (p<0.001, p<0.05, p<0.01, and p<0.001, respectively). Figure 2 and Figure 3 show the depressive and generalized anxiety symptoms changes in both groups.

**Table 1:** Demographic details of the participants BMI: body mass index

Parameters	Yoga group (n=48)	Control group (n=46)
Age (years)	13.44±0.61	13.54±0.64
BMI (kg/m^2^)	18.4±3.67	18.52±3.15
Female	23 (47.91%)	24 (52.17%)
Male	25 (52.08%)	22 (47.82%)
Highest qualification of the parents		
Undergraduate	16 (33.34%)	14 (30.43%)
Graduation	30 (62.5%)	24 (52.17%)
Post-graduation	3 (6.25%)	1 (2.17%)
Which is the best mode of learning for you?		
Combination of online and offline	11 (22.91%)	7 (15.21%)
Offline	34 (70.83%)	36 (78.26%)
Online	3 (6.25%)	3 (6.52%)

Repeated measures ANOVA showed statistically significant changes in SAS-SV scores only, not in other parameters. The f-values of SAS-SV were states (state = F(1,93) = 71.97; p<0.001, η2=0.44), group (group = F(1,93) = 23.38; p<0.001, η2=0.2), and states*group (state*group = F(1,93) = 25.78; p<0.001, η2=0.22). Post hoc analysis with Bonferroni correction showed that the yoga group had statistically significant changes. Furthermore, the yoga group had a higher magnitude of change that is reported by effect size and percentage change, which is displayed in Table 2.

**Table 2:** Mean±SD of the scores of different parameters *p<0.05, ***p<0.001 SAS-SV: Smartphone Addiction Scale-Short Version, WHO-5: World Health Organization Well-Being Index, PHQ-9: Patient Health Questionnaire-9, GAD-7: Generalized Anxiety Disorder-7, SD: standard deviation, %age: percentage

Parameters	Yoga group				Control group			
	Pre	Post	%age change	Effect size	Pre	Post	%age change	Effect size
SAS-SV	39±10.66	26.44±9.05***	-32.18	1.26	26.15±9.3	23±8.19*	-12.05	0.36
WHO-5	16.77±3.97	17.65±3.97	5.23	0.22	17.67±3.35	17.84±4.18	0.98	0.05
PHQ-9	11.34±3.68	7.42±4.66***	-34.53	0.92	9.41±4.4	8.21±5.87	-12.70	0.23
GAD-7	7.83±3.06	6.32±3.78*	-20.57	0.47	7.89±4.81	7.06±5.31	-10.47	0.16

Similarly, regarding screen time, YG had a higher magnitude of decreased time spent on total weekdays, total weeknights, and total weekends (p<0.001, p<0.01, and p<0.001, respectively). Furthermore, in the yoga group, the majority of the sub-factors of screen time changes were statistically significant. Additionally, time spent on television showed a constant decrease in all modes such as weekdays, weeknights, and weekends. The results of screen time are shown in Table 3. In some of the questionnaires, such as SAS-SV, GAD-7, and some sub-factors of screen time, there were baseline differences. Hence, to rule out that the changes were not due to the baseline differences, effect size (magnitude of change) and percentage changes were calculated for all the parameters.

**Table 3:** Mean±SD of screen time *p<0.05, **p<0.01, ***p<0.001 SD: standard deviation, %age: percentage, TV: television

Factor and subfactor	Yoga group					Control group			
	Pre	Post	%age change	Effect size	Pre		Post	%age change	Effect size
TV	2.57±1.7	1.69±1.33**	-34	0.56	2.43±1.92		1.9±1.11	-22	0.32
TV-connected devices	1.16±1.58	0.67±1.02*	-41	0.34	0.66±1.41		0.54±0.84	-18	0.10
Laptop computer	1.1±1.5	0.75±1.08	-32	0.26	0.76±1.09		0.54±0.93	-30	0.22
Smartphone	2.7±1.94	1.78±1.14**	-34	0.54	1.93±1.38		1.22±1.12**	-37	0.56
Tablet	0.69±1.45	0.32±0.91**	-53	0.29	0.34±1.12		0.26±0.97	-22	0.07
Weekdays total	8.24±4.29	5.24±3.36***	-36	0.77	6.15±3.87		4.49±3.05**	-27	0.47
TV	2.21±1.59	1.24±1.32***	-44	0.66	1.73±1.4		1.46±1.41	-16	0.19
TV-connected devices	1±1.47	0.37±0.93	-63	0.49	0.18±0.69		0.32±0.63**	77	0.22
Laptop computer	0.86±1.51	0.59±0.88	-32	0.21	0.47±0.98		0.49±0.79	4	0.02
Smartphone	1.65±2.19	1.33±1.06	-19	0.17	1.35±1.54		1.02±0.95	-24	0.24
Tablet	0.47±1	0.12±0.34*	-74	0.39	0.05±0.26		0.39±1.12*	600	0.34
Weeknight total	6.21±4.5	3.66±3.01**	-41	0.64	3.8±2.81		3.71±3.37	-3	0.03
TV	3.67±2.3	2.74±1.97*	-25	0.43	2.8±1.75		2.33±1.63	-17	0.28
TV-connected devices	1.62±1.86	0.6±1.31***	-62	0.61	0.67±1.49		0.59±1.21	-12	0.06
Laptop computer	1.41±2.16	1.04±1.22	-26	0.19	1.17±1.78		0.48±0.95*	-59	0.45
Smartphone	2.94±2.47	2.21±1.62*	-25	0.34	2.47±1.88		1.89±1.6	-23	0.32
Tablet	0.47±1.09	0.15±0.42*	-68	0.34	0.06±0.33		0.3±0.93*	344	0.29
Weekend total	10.13±4.99	6.76±4.49***	-33	0.71	7.2±4.76		5.61±3.62*	-22	0.37
Weekdays	3.62±1.87	1.89±1.45***	-48	1.02	2.73±1.47		1.66±1.07***	-39	0.81
Weeknight	1.86±1.85	1.3±0.88	-30	0.35	2.2±1.61		1.33±1.06**	-40	0.62
Weekend	2.99±2.45	2.85±1.84	-5	0.06	3.15±2.12		2.64±1.78	-16	0.26
Background screen total	8.48±4.52	6.04±3.19**	-29	0.61	8.09±3.88		5.64±2.9**	-30	0.70

Additionally, Table 4 shows the correlation coefficient values of different psychological parameters. Only those parameters where there was statistical significance have been reported in the table. PHQ had a high-level positive correlation with GAD-7 (r=0.64, p<0.01), while PHQ and GAD-7 both had a medium-level positive correlation with SAS-SV (r=0.37, p<0.01, and r=0.34, p<0.01, respectively). In contrast, both PHQ and GAD-7 had a medium-level negative correlation with WHO-5 (p<0.01).

**Table 4:** Correlation coefficient values of different parameters *p<0.05, **p<0.01 PHQ-9: Patient Health Questionnaire-9, GAD-7: Generalized Anxiety Disorder-7, WHO-5: World Health Organization (WHO)-5, SAS-SV: Smartphone Addiction Scale-Short Version

Correlation coefficient	PHQ-9	GAD-7	WHO-5	SAS-SV
PHQ-9	1	0.64^**^	-0.30^**^	0.37^**^
GAD-7	0.64^**^	1	-0.42^**^	0.34^**^

## Discussion

The current study examined the effects of yoga practice on screen time consumption, smartphone addiction, generalized anxiety, depression, and quality of life in adolescents. A decrease in generalized anxiety and depressive symptoms and screen time was observed following yoga practice in students. These findings indicate that yoga therapy effectively reduces screen consumption and mitigates the complications of electronic gadgets' overuse.

The reduction in the time spent on the screen of any electronic gadgets after the yoga practice is a significant finding of the current study. These findings highlight that the reduction in screen time was mainly observed for TV and TV-connected devices during weekdays, weeknights, and weekends. While a decrease in screen time for mobile and tablets was observed only during the weekends. TV is the most accessible electronic device for students; moreover, TV is less of a private device. Therefore, it is very easy to get on TV and TV-connected devices. On the other hand, students might have restricted access to smartphones and tablets, as many parents provide limited access to the devices. In light of these factors, differences in the benefits of yoga practice for different devices and days are understandable.

Recently, a study by Tadpatrikar et al. explored the effect of psychotherapy and yoga as adjuvant therapy on the excessive use of technology and the internet. The study reported that it effectively reduced the symptoms and severity of technology addiction in young adult subjects [20]. The current research has attempted to explore the integrated approach of yoga modules for addressing excessive screen time use in adolescents aged 13-17. The findings of this study report that excessive screen time use was significantly reduced in students who practice yoga. This is an important finding, as merely reducing screen time can help prevent many physiological and psychological problems. Excessive screen time is known to lead to a range of adverse health effects, impacting both physical and mental well-being, as mentioned above [3,4,6,7].

Apart from the reduction in screen time, the current study also found that the SAS-SV, generalized anxiety, and depressive symptoms were also reduced after the practice of yoga. These results align with previous studies' findings that have demonstrated a reduction in stress, depressive, and anxiety symptoms and improved sleep quality, mood, and well-being following yoga practice. For instance, a regular practice of yoga has been shown to have a positive influence on dysphoric moods, emotional regulation, and self-esteem [21]. Similarly, another study has reported improved academic performance and executive functions following yoga practice [22].

The practice of yoga requires concentration to balance and coordinate the movements of postures to synchronize with breath patterns that facilitate attentional enhancement [23]. Eventually, with regular practice, a habit of being aware of the present moment is cultivated, which might contribute to increased mindfulness. Increased mindfulness has cognitive and psychological effects in terms of improved attention, executive function, reduced stress, and anxiety levels [24]. For example, a four-year qualitative study was conducted on developing mindfulness among college students for self-care by engaging them in a 15-week hatha yoga, meditation, qigong, and mindfulness-based stress reduction practices. This study reported that it promoted positive results in their physical, emotional, mental, spiritual, and interpersonal health [25].

Nonetheless, the current study's findings can be attributed to the very nature of the yoga practice, which gets translated into various health benefits. In addition to this, studies report that yoga enhances mindfulness and self-awareness; consequently, it may be possible that continuous yoga practice may develop greater cognitive flexibility, strengthening the ability for self-regulation [26] and control over impulsive behavior. Although speculation, in the current study, the children might have developed self-regulation and cognitive flexibility due to yoga practice, which might have decreased screen time use, SAS-SV scores, anxiety, and depression of higher magnitude among the yoga group.

The major limitation of the current study is the lack of an active control group; rather, an attentional group was provided, i.e., an educational program on digital hygiene was provided to the control group. The time duration of the pre-data collection was very close to the student's academic final examination, which might have affected the data; hence, there were pre-data differences in some parameters.

## Conclusions

The decrease in screen time use, depression, anxiety, and SAS-SV suggests that the practice of yoga can help counteract the sedentary and passive behaviors encouraged by screen time, fostering healthier habits and supporting the proper development of children. Furthermore, it indicates that yoga module practice may enhance cognitive flexibility and self-regulation, leading to a positive influence on screen usage behavior and minimizing health-related complications. More studies are required to understand if yoga can help mitigate the pleasure-seeking behavior that drives the excessive usage of screens.
